# Tumor cell-expressed SerpinB2 is present on microparticles and inhibits metastasis

**DOI:** 10.1002/cam4.229

**Published:** 2014-03-19

**Authors:** Wayne A Schroder, Lee D Major, Thuy T Le, Joy Gardner, Matthew J Sweet, Sabina Janciauskiene, Andreas Suhrbier

**Affiliations:** 1 Inflammation Biology Group, QIMR Berghofer Medical Research InstituteBrisbane, Queensland, 4029, Australia; 2 Institute for Molecular Bioscience, University of QueenslandSt Lucia, 4072, Australia; 3 Department of Respiratory Medicine, Hannover Medical SchoolHannover, 30625, Germany; 4 School of Biomolecular and Physical Sciences, Griffith UniversityNathan, Queensland, 4111, Australia

**Keywords:** Annexin, metastasis, microparticle, phosphatidylserine, SerpinB2

## Abstract

Expression of SerpinB2 (plasminogen activator inhibitor type 2/PAI-2) by certain cancers is associated with a favorable prognosis. Although tumor-associated host tissues can express SerpinB2, no significant differences in the growth of a panel of different tumors in SerpinB2^−/−^ and SerpinB2^+/+^ mice were observed. SerpinB2 expression by cancer cells (via lentiviral transduction) also had no significant effect on the growth of panel of mouse and human tumor lines in vivo or in vitro. SerpinB2 expression by cancer cells did, however, significantly reduce the number of metastases in a B16 metastasis model. SerpinB2-expressing B16 cells also showed reduced migration and increased length of invadopodia-like structures, supporting the classical view that that tumor-derived SerpinB2 is inhibiting extracellular urokinase. Importantly, although SerpinB2 is usually poorly secreted, we found that SerpinB2 effectively reaches the extracellular milieu on the surface of 0.5–1 *μ*m microparticles (MPs), where it was able to inhibit urokinase. We also provide evidence that annexins mediate the binding of SerpinB2 to phosphatidylserine, a lipid characteristically exposed on the surface of MPs. The presence of SerpinB2 on the surface of MPs provides a physiological mechanism whereby cancer cell SerpinB2 can reach the extracellular milieu and access urokinase plasminogen activator (uPA). This may then lead to inhibition of metastasis and a favorable prognosis.

## Introduction

SerpinB2 (also known as plasminogen activator inhibitor type 2 or PAI-2) is a member of the clade B or ovalbumin-like serine protease inhibitor (ov-serpin) subgroup of the serpin superfamily. SerpinB2 is expressed by a range of cells including cancer cells, monocyte/macrophages, fibroblasts, endothelial cells, and dendritic cells and is often upregulated during inflammatory conditions 1.

SerpinB2 is well-described as an inhibitor of the extracellular protease urokinase plasminogen activator (uPA) and (to a lesser extent) tissue plasminogen activator (tPA) in vitro, with SerpinB2 acting as a suicide substrate to form a covalent SerpinB2-uPA or SerpinB2-tPA complex. However, despite >970 publications in PubMed on SerpinB2/PAI-2, evidence that this represents its physiological function in vivo has remained remarkably elusive, with a bewildering array of diverse uPA-dependent and -independent functions and activities attributed to SerpinB2 1. In addition, SerpinB2^−/−^ mice show no obvious defects in plasminogen activation, and mice deficient in both SerpinB2, and PAI type 1 (PAI-1) show no additional plasminogen activation defects over PAI-1^−/−^ mice 2. SerpinB2, like other ov-serpins, lacks a classical secretory signal peptide and is usually inefficiently secreted. Nevertheless, the nonglycosylated 47 kD protein and 58–70 kD glycosylated species are found in the extracellular milieu 3, with endoplasmic reticulum-golgi-independent secretion of SerpinB2 also reported 4. Whether, when, where, and under what conditions uPA/SerpinB2 complexes might form in vivo remains unclear. In contrast, uPA–PAI-1 complexes have been readily detected in inter alia breast cancer samples 5.

SerpinB2 is expressed in a number of different tumors, and SerpinB2 expression in tumor tissues has been associated with favorable prognosis for a number of cancers including breast and pancreatic cancers 6. However, for endometrial, ovarian, and colorectal cancers, SerpinB2 expression is associated with poor prognosis 7. In these studies it is often unclear whether the SerpinB2 is expressed by the transformed cells or by tumor-infiltrating host cells, although it is clear that tumor cells can express SerpinB2 6,8. Whether tumor-cell SerpinB2 is mutated or functionally inactive is also unclear, although for the cervical cancer cell line, CaSki, SerpinB2 was found to be unmutated and active with respect to uPA inhibition 8. A number of mechanisms whereby SerpinB2 expression by the cancer cells might influence tumorigenesis have been proposed, including inhibition of apoptosis 1,9, growth 10,11, and/or uPA receptor signaling 6. SerpinB2 expression by, for instance, tumor-associated macrophages (TAMs) may also modulate anti-tumor immune responses, given (1) the recent evidence that macrophage SerpinB2 can modulate Th1/Th2 responses 12 and (2) the association of dysregulated SerpinB2 expression with Th1/Th2 perturbations in a number of human diseases 1. Perhaps the best described mechanism whereby SerpinB2 expression by cancer cells might improve prognosis is via reduction of uPA-dependent migration/invasion and metastases 13–15.

In summary, although clear links between tumor SerpinB2 expression and cancer prognosis have been established, the cells and mechanism(s) involved remain poorly understood. Herein, we use SerpinB2^−/−^ mice to explore the role of host cell SerpinB2 expression, and use lentiviral transduction of syngeneic tumor cell lines to explore the role of murine SerpinB2 in immunologically intact mice.

## Material and Methods

### Tumor lines

The mouse tumor lines, B16 melanoma (ATCC CRL-6322), B16 cells expressing ovalbumin (B16-OVA) (provided by Dr. K. Rock, Dana-Farber Cancer Institute), Lewis Lung carcinoma (ATCC CRL-1642), RM1 prostate cancer, MC38 colon adenocarcinoma, and Tubo mammary lobular carcinoma 16, were grown as described 17; the latter three lines were kindly provided by Prof. M. Smyth (Peter MacCallum Cancer Centre, Melbourne, Australia). The human cervical cancer, CaSki (ATCC CRL-1550) and histiocytic lymphoma, U937 (ATCC CRL-1593.2) lines were grown as described 8. Cell lines were obtained within the last 10 years and passaged no more than five times, and were tested within the last year using the Promega StemElite ID system (Promega, Alexandria, NSW, Australia) using a minimum of eight STR loci as per ATCC standard ASN-0002.

### Mice, tumor inoculation, and monitoring

SerpinB2^−/−^ and control SerpinB2^+/+^ mice backcrossed 12 times onto the C57BL/6 background were generated as described 12. Tumor cells were trypsinized, washed once in Roswell Park Memorial Institute 1640 medium (RPMI 1640) supplemented with 10% fetal calf serum (FCS), resuspended in RPMI 1640, and 100 *μ*L inoculated s.c. onto the backs of 6- to 8-week-old female mice (5 × 10^5^ B16, 4 × 10^6^ B16-OVA, 10^5^ Tubo, 4 × 10^6^ Lewis Lung, 3 × 10^5^ RM1 or MC38 cells). The width and breadth of each tumor was measured at the indicated times using digital calipers and was expressed as area (width × breadth) 17. The mice were euthanized when tumors reached 100 mm^2^. C57BL/6 and Balb/c mice were obtained from ARC, Perth, Australia.

### Mouse vaccination and challenge

Female SerpinB2^−/−^ or SerpinB2^+/+^mice (6- to 8-week-old) were vaccinated once s.c. with 100 *μ*g SIINFEKL peptide dissolved in RPMI 1640 formulated in complete Freunds adjuvant (CFA) (Sigma Aldrich, Castle Hill, NSW, Australia) (1:1 v:v, final volume 50 *μ*L). After 3 weeks mice were challenged s.c. with 10^5^ B16-OVA and tumor growth monitored as described 17.

### B16 lung metastasis

For lung metastases 10^5^ B16, B16-Control, or B16-SerpinB2 cells were inoculated i.v. in 200 *μ*L RPMI 1640 into 16- to 20-week-old female mice; the mice were then euthanized after 18 days. Lung metastases (clearly visible as black spots) were then counted using a stereo dissecting microscope (10× magnification).

### Ethics statement

All mouse experiments were approved by the QIMR animal ethics committee and adhered to the National Health and Medical Research Council (Australia) code of practice for the care and use of animals.

### Lentivirus vectors and generation of SerpinB2 expressing tumor lines

The plasmids pLOX-CW-EGFP (control), encoding enhanced green florescence protein from a CMV promoter, and pLOX-CWmPAI-2, encoding murine SerpinB2 from a CMV promoter, were constructed as described 18 and kindly provided by Dr. R. J. Fish (University Geneva, Switzerland). Lentiviral vectors were generated as described 18. Tubo cells were transduced with these vectors (transduction efficiency >95%) to generate Tubo-Control and Tubo-SerpinB2.

B16 cells were transduced with a lentivirus vector encoding ZsGreen, or murine SerpinB2 and ZsGreen; ZsGreen being expressed from an internal ribosome entry site. pLVX-mSerpinB2-IRES-ZsGreen1 was constructed by polymerase chain reaction (PCR) amplifying murine SerpinB2 from pLOX-CWmPAI-2 18 and cloning it into pLVX-IRES-ZsGreen1 (Clontech, Mountain View, CA). pLVX-mSerpinB2-IRES-ZsGreen1 and pLVX-IRES-ZsGreen1 were then used to make lentiviral vectors as described previously 18. These lentiviral vectors were used to transduce B16 cells. After 7 days culture, the cells were subject to fluorescence-activated cell sorting (FACS) 19 to generate B16-SerpinB2 and B16-Control cell lines, respectively. The lines were >99% ZsGreen^hi^.

### Immunoblotting analysis

Immunoblotting was performed as described 12 using anti-human SerpinB2 mouse monoclonal antibody (American Diagnostica, Lexington, MA; #3750), an anti-mouse SerpinB2 antibody generated in-house 12 (currently available commercial anti-SerpinB2 antibodies do not recognize murine SerpinB2, data not shown), anti-cleaved Poly (ADP-ribose) polymerase (PARP) (Cell Signaling Technology, Danvers, MA; Asp214), rabbit anti-mouse uPA (Abcam, Waterloo, NSW, Australia; ab20789, 1:5000), rabbit anti-actin (Sigma Aldrich, #AC40), and anti-GAPDH (Millipore, Billerica, MA; MAB 374). Detection was performed by HRP-conjugated sheep anti-mouse (Chemicon, Temecula, CA; #AP326P) and Western Lightning enhanced chemiluminescence substrate (Perkin Elmer, Waltham, MA; #NEL101001EA).

### Migration assays and length determination of invadopodia-like structure

For the migration assays cells (10^4^ per well) were seeded into Transwell cell culture inserts, 8 *μ*m pore size (BD Biosciences, San Jose, CA; #353097) in RPMI 1640 supplemented with 0.1% FCS, with the bottom well containing RPMI 1640 supplemented with 40 *μ*g/mL fibronectin (Sigma Aldrich). The cells were cultured for 2 days and the top cells removed using a cotton wool swab. The cells on the bottom surface were then stained with crystal violet and counted using an inverted light microscope.

For determination of the length of invadopodia-like structures, cells (6 × 10^3^ per well) were seeded into Matrigel (BD Biosciences, #356237) in a 96 well plate (one volume of cells in RPMI 1640 supplemented with 10% FCS to two volumes of Matrigel) and were cultured for 2 days. Cell colonies were then viewed using live cell imaging; Qlympus IX81 microscope (Notting Hill, VIC, Australia) and Cell^M^ software 2.8. The length of invadopodia-like structures was determined from still images.

### Microvesicle preparation and uPA incubation

B16 cells were treated with calcium ionophore (A23187) (Sigma Aldrich) (10 *μ*mol/L) for 30 min at 37°C. U937 cells were treated for 2 days with 50 ng/mL PMA. The supernatants were harvested and cell debris and apoptotic bodies were pelleted by centrifugation at 2000*g* for 10 min to bring down cell debris and apoptotic bodies. The supernatant was then subjected to further centrifugation at 20,000*g* for 30 min to pellet MPs. Exosomes were prepared by further centrifugation of this supernatant at 100,000*g* for 1 h. Pelleted fractions (30 *μ*g per well) were loaded onto SDS-PAGE (sodium dodecyl sulfate polyacrylamide gel electrophoresis) for immunoblotting analysis.

MPs (30 *μ*g) were incubated with murine 20 IU uPA (Innovative Research, Novi, MI) for 20 min at 37°C. The mixtures were then solubilized in SDS-PAGE sample buffer, snap frozen, and stored at −70°C.

### Microvesicle uPA assays

MP and exosome fractions (1.5 *μ*g/*μ*L) resuspended in hydroxyethyl piperazineethanesulfonic acid (HEPES) buffer, phenol red-free RPMI 1640 supplemented with 5% FCS, and 0.1% Triton X-100 were then analyzed for uPA activity using the Mouse uPA Activity Assay kit (Molecular Innovations, Novi, MI; #MUPAKT).

### Confocal microscopy

U937 cells plated on glass 25 mm^2^ coverslips (2.5 × 10^5^ cells/well in a six-well plate) were differentiated with 25 ng/mL PMA for 48 h. Cells were washed and live stained with mouse anti-SerpinB2 (American Diagnostica, #3750) and fluorescein isothiocyanate (FITC)-labeled anti-mouse secondary antibody (Chemicon) at 4°C. Costaining for CD11b used rabbit anti-CD11b (Abcam) and anti-rabbit FITC (Chemicon), and anti-mouse Texas Red-X for detecting the anti-SerpinB2 antibody (Molecular Probes, Eugene, OR). Cells were washed at 4°C and fixed in 4% paraformaldehyde. Coverslips were mounted onto slides using ProLong Gold (Invitrogen, Carlsbad, CA) and analyzed on a Leica TCS confocal microscope (Leica Microsystems, North Ryde, NSW, Australia).

### FACS analysis

FACS analysis was performed using the FACS LSR Fortessa (BD Biosciences) and FlowJo software (Tree Star, Ashland, OR), FITC-labeled annexin V (BD Biosciences, #556420), anti-human PAI-2 monoclonal antibody (IgG2a) (American Diagnostica, #3750) with secondary anti-mouse Cy3 (Jackson Labs, Bar Harbor, ME; #115-165-146) (isotype control, anti-C/EBP*β*, H7, Santa Cruz, Dallas, TX), and goat anti-human SerpinB2 (American Diagnostica, #375G) with secondary APC-labeled donkey anti-goat (R&D Systems, Minneapolis, MN, #F0108) (control; goat anti-E7, #sc-7962, Santa Cruz). Dual labeling using anti-SerpinB2 antibodies and annexin V was undertaken at 4°C by labeling first with anti-SerpinB2 antibodies, washing, then the secondary antibody, followed by annexin V.

### Lipid-binding assays

CaSki cells were lysed (10 mmol/L Tris pH 7.5, 10 mmol/L NaCl, 2 mmol/L ethylenediaminetetraacetic acid, 0.5% Triton X-100, Complete Protease Inhibitor Cocktail [Roche, Brisbane, Qld, Australia]) and cell debris removed by centrifugation at 2000*g* for 10 min. Lipid strips (Echelon Biosciences Inc., Salt Lake City, UT; #P-6002) were blocked for 2 h at room temperature with tris buffered saline (TBS) (137 mmol/L NaCl, 20 mmol/L Tris pH 7.6) with 3% bovine serum albumin, and were then incubated with CaSki lysates (1 mg/mL) in the presence of annexin-binding buffer (10 mmol/L HEPES, 140 mmol/L NaCl, 2.5 mmol/L CaCl_2_) overnight at 4°C. The strips were washed in TBS with 0.1% Tween20, probed with anti-human SerpinB2 antibody (American Diagnostica, #375G) and anti-goat HRP.

HEK293 cells were transfected (GeneJammer; Agilent Tech., Victoria, Australia) with DNA plasmids encoding EGFP-SerpinB2 or EGFP. Lysates were prepared and incubated with lipid strips as above. Anti-EGFP antibody (Invitrogen, #A6455) and secondary anti-rabbit HRP was used to detect bound SerpinB2.

Phosphatidylserine (PS) beads (Echelon; #P-B0Ps) (50 *μ*L) were incubated with 0.5 *μ*g recombinant human SerpinB2 (ProSci Inc., Poway, CA) with and without 0.5 *μ*g recombinant annexin I (Prospec, East Brunswick, NJ) overnight at 4°C in annexin-binding buffer, and washed four times in PS bead wash buffer (10 mmol/L HEPES pH 7.4, 150 mmol/L NaCl, 0.25% NP40). Finally the beads were boiled in SDS sample buffer and bead-bound proteins resolved by SDS-PAGE followed by immunoblotting analysis with anti-SerpinB2 antibody (American Diagnostica, #375G) and anti-goat HRP.

### Statistics

Statistical analysis was performed using SPSS for Windows (version 15.0, 2007; SPSS, Chicago, IL). For comparison of two samples, the *t*-test was used when the difference in the variances was <4 and skewness was >−2 and kurtosis was <2. The Mann–Whitney *U* test was used if variance differences were <4 and skewness was <−2 or kurtosis was >2, otherwise the Kolmogorov–Smirnov test was used.

## Results

### Host SerpinB2 expression had no significant effect on tumor growth

**Figure 1:**
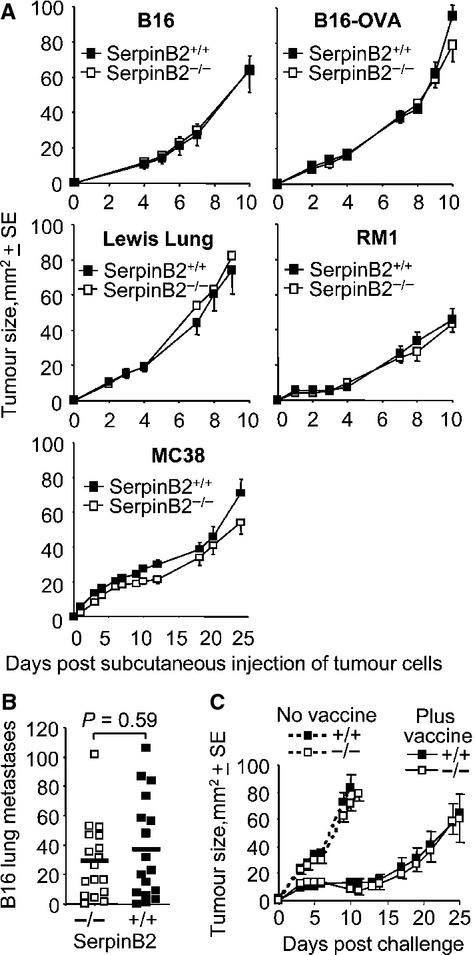
Tumor growth in SerpinB2^−/−^ and SerpinB2^+/+^ mice. (A) Groups (*n* = 5–8 per group) of age-matched SerpinB2^+/+^ and SerpinB2^−/−^ mice were injected s.c. with different tumors and their growth monitored over time. B16, melanoma; B16-OVA, B16 stably expressing ovalbumin, Lewis lung carcinoma; RM1, prostate cancer; MC38, colon carcinoma. (B) SerpinB2^−/−^ and SerpinB2^+/+^ mice (as above, *n* = 17 per group) were injected i.v. with B16 cells and after 18 days lung metastases were counted. Statistics by Mann–Whitney *U* test. (C) SerpinB2^−/−^ and SerpinB2^+/+^ mice (*n* = 7–8 per group) were not vaccinated (no vaccine) or were vaccinated with SIINFEKL formulated in CFA (plus vaccine). After 14 days mice were challenged with B16-OVA and tumor growth monitored over time.

Macrophages, fibroblasts, and endothelial cells all express SerpinB2 1, and these cells have also been shown to express SerpinB2 when associated with tumors in vivo 20–22. To determine whether host SerpinB2 expression influences tumor growth, a panel of syngeneic tumor lines were grown subcutaneously in SerpinB2^−/−^ and SerpinB2^+/+^ mice. None of the four tumor lines tested showed any difference in growth in the two strains (Fig. 1A). To determine whether host SerpinB2 would influence formation of metastases, B16 cells were injected i.v. and lung metastases counted after 18 days 17. No significant differences in the number of metastases between SerpinB2^−/−^ and SerpinB2^+/+^ mice were observed (Fig. 1B). Thus, host cell SerpinB2 expression appears to have no significant effect on tumor growth or establishment of metastases.

### Anti-cancer CD8 responses and tumor Th1/Th2 markers in SerpinB2^−/−^ mice

Protein immunization using CFA in SerpinB2^−/−^ mice resulted in increased Th1 CD4^+^ T-cell and antibody responses 12. To determine the effect of SerpinB2 expression on anti-cancer CD8 T-cell induction, SerpinB2^−/−^ and SerpinB2^+/+^ mice were immunized with CFA containing SIINFEKL, a CD8^+^ T-cell epitope specific for ovalbumin 17. The mice were then challenged with B16-OVA and tumor growth monitored. No difference in B16-OVA growth in SerpinB2^−/−^ and SerpinB2^+/+^ mice was observed (Fig. 1C). Similar results were obtained when animals were immunized with epitopes from endogenous B16 antigens (KVPRNQDWL and SVYDFFVWL) 17 followed by challenge with parental B16 cells (data not shown). The Th1 bias previously reported in SerpinB2^−/−^ mice 12 thus did not extend to enhanced anti-cancer CD8^+^ T-cell responses.

The levels of KVPRNQDWL- and SVYDFFVWL-specific CD8^+^ T-cells responses induced following i.v. injection of B16 cells was also not different in SerpinB2^−/−^ and SerpinB2^+/+^ mice (Fig. S1). These data are consistent with Figure 1B, as these CD8^+^ T-cell responses ordinarily reduce the number of metastases in this model 17. There were also no significant differences in TNF, IL-6, TGF-*β*, or Arg-1 mRNA levels in s.c. B16 tumors from SerpinB2^−/−^ and SerpinB2^+/+^ mice (Fig. S2). The previously reported role for SerpinB2 in modulating Th1/Th2 markers after infection or vaccination 12,23,24, thus does not appear to extend to tumor settings.

### The effect of SerpinB2 expression by cancer cells on tumor growth

**Figure 2:**
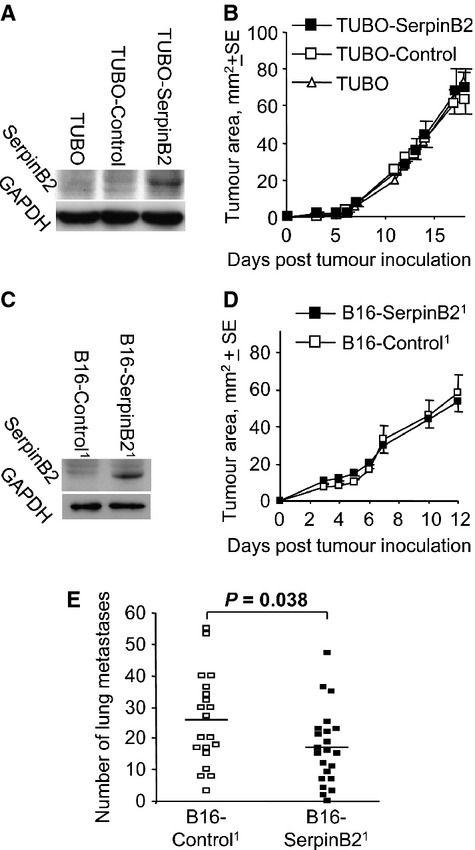
SerpinB2 expression in B16 and Tubo cells and their growth in vivo. (A) Parental Tubo cells (Tubo) or Tubo cells transduced with a lentiviral vector encoding EGFP (Tubo-Control) or SerpinB2 (Tubo-SerpinB2) were analyzed by immunoblotting for murine SerpinB2 expression using anti-murine SerpinB2 antibody. (B) The same cells described in A were inoculated s.c. into Balb/c mice and tumor growth was monitored over time (*n* = 12 mice per group). (C) B16 cells were transduced with a lentiviral vector encoding ZsGreen or SerpinB2-ZsGreen and were then FACs sorted to produce B16-Control and B16-SerpinB2 cell populations, respectively, that were both >99% ZsGreen^hi^. The cell lines were then analyzed by immunoblotting as in A. (D) B16-Control and B16-SerpinB2 (described in C) were inoculated s.c. into C57BL/6 mice and growth monitored over time. (E) SerpinB2 expression by B16 cells reduces metastases. B16-Control and B16-SerpinB2 cells (described in the figure2) were inoculated i.v. into C57BL/6 mice (*n* = 20/22 per group) and on day 18 the number of lung metastases was determined. Statistics by *t*-test.

Cancer cells have been shown to express SerpinB2 6,8 and several studies have suggested that SerpinB2 expression by cancer cells can inhibit their proliferation in vitro 6,10,11,25. To determine whether SerpinB2 expression by cancer cells affects tumor growth in vivo, B16-SerpinB2 and B16-Control, and Tubo-SerpinB2 and Tubo-Control cell lines were generated by lentiviral transduction. We have previously shown that parental B16 and Tubo (breast cancer) cells do not express detectable levels of SerpinB2 mRNA 8. Expression of SerpinB2 protein and mRNA in Tubo-SerpinB2 and B16-SerpinB2 was demonstrated by immunoblotting (Fig. 2A and C) and quantitative reverse transcription (qRT)-PCR (Fig. S3). These cell lines expressed ≈30% of the SerpinB2 mRNA levels seen in resident peritoneal macrophages as assessed by qRT-PCR (Fig. S3). Western analysis showed similar relative SerpinB2 protein expression levels (data not shown). SerpinB2 expression levels in these transduced tumor cell lines were thus not supraphysiological. No methodology currently exists for quantifying (i.e., ng/mg) murine SerpinB2 protein expression levels.

No differences in tumor growth were observed when Tubo-SerpinB2, Tubo-Control, or parental Tubo cells were grown s.c. in syngeneic Balb/c mice (Fig. 2B). Ki67 staining of these tumors by immunohistochemistry also showed no differences in the number of proliferating cells (Fig. S4). There was also no significant difference in the growth of B16-SerpinB2 and B16-Control tumors in C57BL/6 mice (Fig. 2D). The same result was obtained when SerpinB2^−/−^ mice were used (data not shown). qRT-PCR analysis of the tumors in this latter experiment illustrated that SerpinB2 expression in B16-SerpinB2 cells was retained in vivo over the 12–15 days of tumor growth (Fig. S5).

In addition to the in vivo experiments described above, we were also unable to observe a significant effect of SerpinB2 expression on the proliferation of B16 and Tubo lines in vitro (Fig. S6). We also lentivirally transduced a panel of human cancer cell lines with human SerpinB2 and again saw no effect on proliferation or cell cycle in vitro (Fig. S7), consistent with earlier work 8,18. Effects on cell cycle in vitro were also not observed when transient transfection was used to express SerpinB2 in cancer cell lines (Fig. S8). Growth differences were also not apparent when immortalized murine embryonic fibroblasts (MEFs) or macrophages from SerpinB2^+/+^ and SerpinB2^−/−^ mice were tested (Figs. S9 and S10). We were also unable to find evidence for human-SerpinB2 expression in transduced tumor cells protecting the cells from TNF-induced apoptosis (data not shown), also in agreement with earlier work 18.

Taken together these experiments suggest that SerpinB2 expression by transformed cells does not significantly affect tumor growth in vivo or cancer cell proliferation in vitro.

### SerpinB2 expression by B16 cells reduced metastasis

Previous studies reported that SerpinB2 expression by tumor cells reduces metastases 13,15. However, these studies used human cell lines expressing human SerpinB2 in immune-deficient mice. In addition, two of these reports 13,15 used SerpinB2-expressing cell lines generated by transfection and clonal selection, a process that can result in clonal bias 1,6,26. The other report used transfection with adenovirus vectors 14, a methodology that can be affected by adenovirus-associated toxicity 8. To avoid these issues we tested the lentivirally transduced B16-SerpinB2 (expressing murine SerpinB2) and B16-Control lines in immune competent mice.

Mouse SerpinB2-expressing B16 cells were injected i.v. into syngeneic C57BL/6 mice and 18 days later the number of lung metastases was determined. A significant 35% decrease in the number of metastases was seen in mice that received murine SerpinB2-expressing B16 cells when compared with mice receiving B16-Control cells (Fig. 2E). These data thus support previous reports 13–15 that tumor cell-derived SerpinB2 inhibits metastasis.

### SerpinB2 expression reduced migration and length of invadopodia-like structures

**Figure 3:**
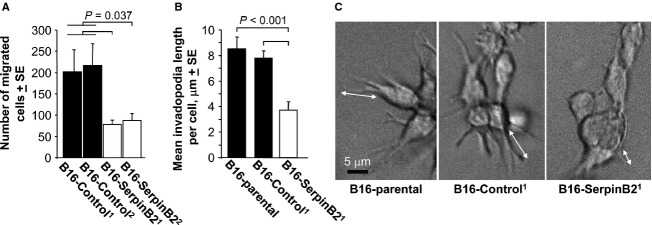
Migration and invasion of SerpinB2-expressing B16 cells. (A) B16-Control and B16-SerpinB2 cell lines (superscript 1 and 2 indicate lines generated on two separate occasions) were seeded into transwells (10^4^ cells/well) and the number of cells that migrated toward 40 *μ*g/mL fibronection determined after 2 days. The data were presented as a mean of three repeat assays, with each bar showing the mean ± SE of the three assays. The B16-Control^1^ and B16-Control^2^ data were combined for statistical analysis (*n* = 6) and were significantly different from both B16-SerpinB2^1^ and B16-SerpinB2^2^ (Statistics by Kolmogorov–Smirnov tests). (B) Parental B16, B16-Control and B16-SerpinB2 cells were seeded into matrigel and after 2 days the cells were examined by live imaging and length of invadopodia-like structures measured using still images (examples shown in C; 22–47 cells were examined per cell line; statistics by *t-*test). (C) Representative images of cells providing the data for B (arrows show examples of length measurements).

As B16 cells express uPA (Fig. S11), the reduced metastasis of B16-SerpinB2 cells (Fig. 2E) may reflect inhibition of uPA-mediated migration 13–15. Using transwell migration assays, the number of the SerpinB2-expressing B16 cells migrating through a cell permeable membrane was shown to be significantly lower when compared with control B16 cells (Fig. 3A). In addition, the length of invadopodia-like structures were significantly reduced in SerpinB2-expressing B16 cells when the cells were seeded into Matrigel (Fig. 3B and C). Invadopodia are actin-rich cell membrane protrusions that are intimately associated with invasion and are frequently seen in metastatic cancer cells 27. When the experiment in Figure 3B and C was repeated and cell actin stained with phalloidin, a similar result was obtained (Fig. S12). These data are consistent with reduced uPA-activated plasmin and matrix metalloproteinase activity, given the extracellular matrix proteolysis mediated by these proteases is believed to be involved in the protrusion of invadopodia 28–31. The number of cells per colony and the number of invadopodia-like structures per cell were not significantly different between the groups (data not shown). The latter suggests SerpinB2 had no significant effect on the formation of these structures.

These data support the classical view 32 that tumor-derived SerpinB2 inhibits uPA-dependent migration and invasion, thereby reducing metastasis and improving prognosis 13–15.

### SerpinB2 and microparticles

SerpinB2 is usually inefficiently secreted 1 and clearly uPA inhibition would require SerpinB2 reaching the extracellular milieu. A recent publication suggested SerpinB2 is present on the surface of syncytiotrophoblast microparticles (MPs) 33. Cancer cells are well known to generate MPs 34, with tumor-derived MPs also shown to contain uPA and to be involved in regulating metastasis 35,36. We thus sought to determine whether SerpinB2 might be associated with MPs in SerpinB2-expressing B16 cells.

**Figure 4:**
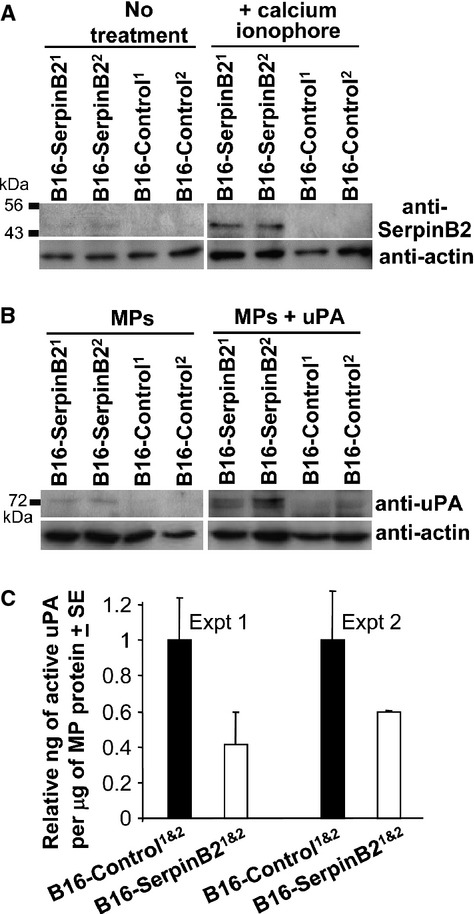
SerpinB2 microparticles. (A) B16-Control and B16-SerpinB2 cell lines were treated without (no treatment) or with calcium ionophore (+calcium ionophore) for 30 min, supernatants were harvested, MPs isolated by differential centrifugation and analyzed by immunoblotting using anti-murine SerpinB2 and anti-actin antibodies. (B) The same MPs described in A were incubated without (MPs) or with recombinant murine uPA (MPs + uPA), solubilized in SDS and analyzed by immunoblotting using anti-murine uPA and anti-actin antibodies. (C) Active uPA levels in MPs from B16-SerpinB2 and B16-Control cells as measured by capture ELISA. Data were presented as relative to the mean uPA levels in MPs from the two B16-SerpinB2 lines. Each bar represents the mean relative uPA level in MPs from the two SerpinB2-expressing or two control lines (*P* = 0.021 for B16-SerpinB2 vs. B16-Control when the two experiments are combined, i.e., *n* = 4 per group).

SerpinB2-expressing and control B16 cells were cultured in the absence or presence of calcium ionophore, a standard agent for inducing production of MPs 37. The MPs were then isolated from culture supernatants by differential centrifugation and analyzed by immunoblotting. In the absence of calcium ionophore only a hint of SerpinB2 could be seen in MPs derived from SerpinB2-expressing B16 cell supernatants (Fig. 4A, No treatment, B16-SerpinB2), with no SerpinB2 detected in MPs from B16-Control cells (Fig. 4B, No treatment, B16-Control). After 30 min of treatment with calcium ionophore, the presence of SerpinB2 protein could be clearly detected in MPs from SerpinB2-expressing B16 cell supernatants (B16-SerpinB2^1 & 2^), but not in MPs from control B16 lines (B16-Control^1 & 2^) (Fig. 4A, +calcium ionophore). No SerpinB2 was detected in exosome fractions from SerpinB2-expressing B16 cells (data not shown).

To ascertain whether MP-associated SerpinB2 had bound endogenous uPA, the MPs were analyzed by immunoblotting using anti-uPA antibody. Only faint anti-uPA antibody-reactive ≈75 kDa bands (indicative of covalent uPA/SerpinB2 complexes) could be seen in MPs from SerpinB2-expressing B16 cells, but not B16-Control cells (Fig. 4B, MPs). The MPs were incubated with recombinant murine uPA for 30 min and were then solubilized in SDS-PAGE buffer. SDS prevents inhibition of uPA by SerpinB2 38, so only surface bound SerpinB2 would interact with uPA in this assay. Clear ≈75 kDa bands were detected in uPA-treated MPs from SerpinB2-expressing B16 cells, but not in uPA-treated MPs from B16-Control cells (Fig. 4B, MPs + uPA).

These results illustrate that SerpinB2 is present on MPs derived from B16-SerpinB2 cells and suggests that MP-associated SerpinB2 is able to inhibit uPA present in the extracellular milieu.

### uPA activity on MP from B16-SerpinB2 and B16-Control cells

To determine whether MP-associated uPA activity was reduced in MPs from B16-SerpinB2 cells, a commercial capture the enzyme-linked immunosorbent assay (ELISA) that measures active uPA was used. MPs from B16-SerpinB2 cell lines showed ≈40–60% lower activity than MPs from B16-Control lines (Fig. 4C). uPA activity from exosome fractions was low and not significantly different between SerpinB2-expressing and control cells (data not shown).

### SerpinB2 is associated with MPs derived from U937 human monocytes

**Figure 5:**
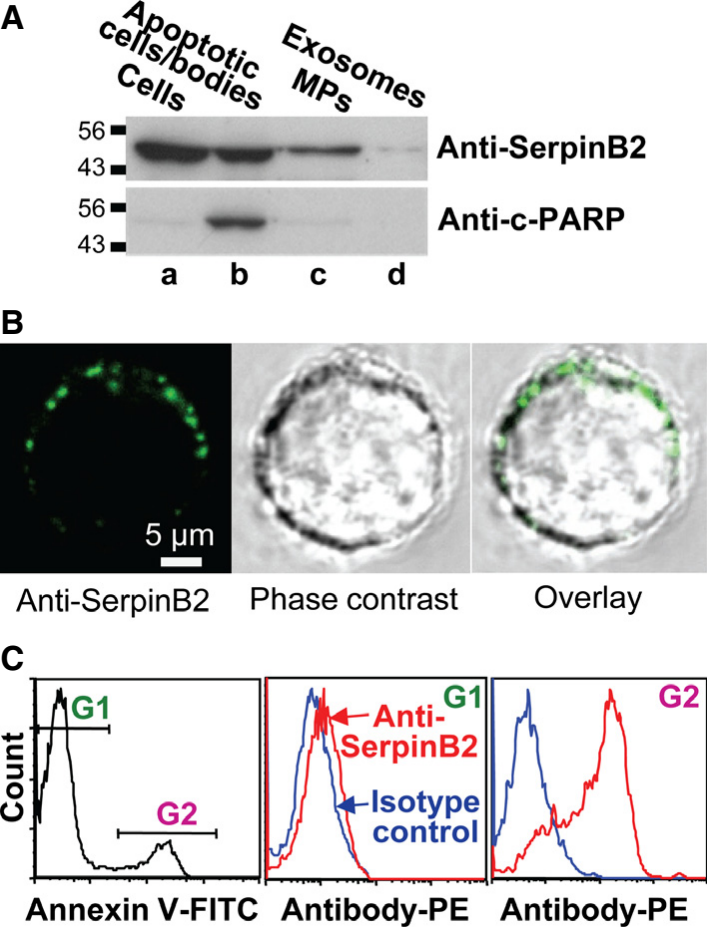
(A) U937 cells were treated with PMA (25 ng/mL for 2 days) and calcium ionophore (10 *μ*mol/L for 30 min). Whole cell lysate (lane a), apoptotic cells/apoptotic bodies (obtained from the culture supernatant by centrifugation at 2000*g* for 10 min) (lane b), the MP fraction (obtained from the latter supernatant by further centrifugation at 20000*g* for 30 min) (lane c), and the exosome fraction (obtained from the latter supernatant by further centrifugation at 100,000*g* for 1 h) (lane d) were analyzed by immunoblotting (10 *μ*g of protein loaded per lane). The antibodies used were an anti-human SerpinB2 monoclonal antibody and anticleaved PARP antibody (c-PARP) (a marker of apoptosis). (B) U937 cells were treated with PMA and calcium ionophore as above and were then live stained at 4°C and viewed by confocal microscopy. Propidium iodide was included with the FITC-labeled secondary antibody to identify and exclude permeabilized cells. Top row; staining with anti-human SerpinB2 monoclonal antibody showing representative fluorescence image (left), phase-contrast image (middle), and overly (right). Bottom row; costaining with the anti-human SerpinB2 and anti-CD11b antibodies. (C) U937 cells treated with PMA and calcium ionophore as above, were stained with annexin V-FITC (to detect PS) and anti-human SerpinB2 monoclonal antibody (with PE-labeled secondary). FACS analysis showed an annexin V^low^ population gated as G1, and an annexin V^hi^ population gated as G2 (left panel). The G1 population showed only marginal staining with anti-SerpinB2 antibody (middle panel). The G2 population was clearly stained with the anti-SerpinB2 antibody (right panel). The PS^hi^ population was thus also SerpinB2^hi^.

Phorbol ester treatment of the human histiocytic lymphoma cell line, U937, represents a well-recognized system for studying SerpinB2 secretion 32. U937 cells were treated with PMA for 2 days and calcium ionophore for 30 min and apoptotic cells/bodies, MPs, and exosomes isolated from the supernatant by differential centrifugation. SerpinB2 was clearly present in the apoptotic cells/bodies (which expressed the apoptotic marker, cleaved PARP, c-PARP) and MP fractions, with the exosome fraction containing comparatively much lower levels (Fig. 5A).

To obtain further evidence that SerpinB2 is externalized on MPs, U937 cells were treated for 2 days with 25 ng/mL PMA and calcium ionophore for 30 min and were then live stained with an anti-human SerpinB2 monoclonal antibody. SerpinB2 staining of 0.5–1 *μ*m diameter spherical bodies associated with the plasma membrane was clearly apparent by confocal microscopy (Fig. 5B). No staining of the cell interior (where SerpinB2 is abundantly expressed; Fig. 5A) 32 was observed (Fig. 5B), illustrating the plasma membrane was intact. In addition, FACS analysis of these live cells showed two populations; one with low surface expression of PS (Fig. 5C, left panel, G1) and a subpopulation with high surface PS expression (Fig. 5C, left panel, G21). (Permeabilized cells staining positive with propidium iodide were excluded.) The translocation of PS from the inner to the outer side of the plasma membrane is a characteristic event during MP formation 39. Importantly, the PS^low^ population did not show significant levels of SerpinB2 staining (Fig. 5C, middle panel, G1), whereas the PS^hi^ population clearly showed high levels of SerpinB2 staining (Fig. 5C, right panel, G2). Essentially the same results were obtained when a goat polyclonal anti-human SerpinB2 antibody was used (data not shown). These data support the view that MPs express SerpinB2 on their surface.

### SerpinB2 and annexin binding

SerpinB2 has been reported to bind a bewildering variety of proteins 1. However, of these, the annexins would be clear candidates for mediating association of SerpinB2 with MPs as (1) SerpinB2 was reported to bind multiple annexins 40,41, (2) several annexins are present on MPs 42,43, and (3) all annexins are known to bind PS, a lipid characteristically present on the surface of MPs 39.

**Figure 6:**
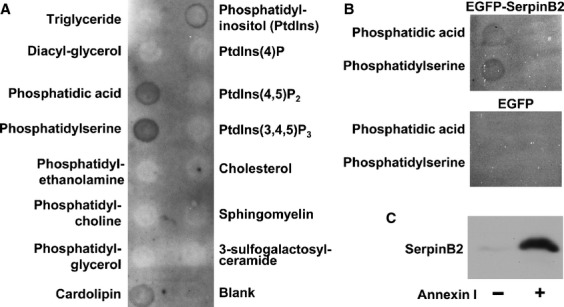
SerpinB2 association with PS via annexin. (A) CaSki cell lysates were incubated with membranes arrayed with the indicated lipids, the membranes were washed and probed with anti-human polyclonal SerpinB2 antibody. (B) HEK293 cells were transiently transfected with DNA plasmids encoding EGFP-SerpinB2 or EGFP, and cell lysates incubated with the lipid membranes as for A. SerpinB2 was detected using anti-EGFP antibody. (C) PS beads were incubated with purified recombinant human SerpinB2 in the absence (−) or presence (+) of purified recombinant human annexin I. The beads were washed, and bead-bound proteins solubilized in SDS and analyzed by immunoblotting using anti-human SerpinB2 antibody.

To determine whether endogenously produced SerpinB2 associates with PS, lysates of CaSki cells were incubated with membranes arrayed with a range of lipids. CaSki cells constitutively make high levels of wild-type human SerpinB2 8. Anti-human SerpinB2 antibody was then used to detect membrane-bound SerpinB2 using standard immunoblotting protocols. SerpinB2 clearly associated with PS and (less consistently) with phosphatidic acid (Figs. 6A and S13), a phospholipid to which some annexins also bind 44. Using a different SerpinB2 expression system (lysates of HEK293T cells transiently transfected with EGFP-SerpinB2) and detection antibody (anti-EGFP antibody), EGFP-SerpinB2 (but not EGFP) was again shown to associate with PS and (to a lesser extent) phosphatidic acid (Fig. 6B). These experiments clearly show that SerpinB2 associates with PS; however, recombinant SerpinB2 alone did not associate with any lipids (data not shown).

To illustrate that annexin is required for the association of SerpinB2 with PS, PS agarose beads were mixed with purified recombinant human SerpinB2 and recombinant human annexin I. SerpinB2 was clearly shown to associate with PS in the presence, but not the absence, of annexin I (Fig. 6C), illustrating that annexin is required and sufficient for the association of SerpinB2 with PS.

## Discussion

Herein, we show that SerpinB2 deficiency in host tissues had no significant effect on the growth of four different tumors types in vivo (Fig. 1). A similar conclusion was reported for host PAI-1 expression 45. There was also no significant effect of host SerpinB2 expression on Th1/Th2 responses and anti-cancer immunity. In tumor settings, SerpinB2 expression in host tissues may have little influence or may simply be too low to mediate significant effects. Low-SerpinB2 expression by TAMs has been reported previously 20, and no correlation was seen between SerpinB2 expression by TAMs and survival 20.

Previous in vitro work suggested a role for tumor cell-expressed SerpinB2 in suppressing tumor cell growth 6,10,11,25. However, we were unable, in several settings, to observe any significant effects of SerpinB2 expression on cell growth or cell cycle in vitro, or cancer growth in vivo. This discrepancy may reflect the use in previous studies of transfection and clonal selection to generate SerpinB2-expressing tumor cell lines. Data obtained from clonally selected lines are potentially compromised by clonal bias 6,26, an issue avoided by lentiviral transduction 1. Resistance to apoptosis was also seen in SerpinB2-expressing cells after transfection and clonal selection, but not in the same cells expressing SerpinB2 after lentiviral transduction 18. Another explanation for the discrepancy may be that, in specific settings, SerpinB2 expression affects differentiation, which in turn leads to growth changes 11,25. In conclusion, although we have not explored all possible tumor types and/or settings, our data does not support the view that SerpinB2 expression by cancer cells affects their growth directly.

A significant 35% reduction in metastases was observed after i.v. injection of lentivirally transduced B16 cells expressing SerpinB2 (Fig. 2C). These data are consistent with previous reports 13,14, with Praus et al. 14 showing a comparable reduction (22–50%) in the number of metastases in nude mice after implantation of the human fibrosarcoma line, HT1080, expressing human SerpinB2 via adenoviral transduction. We also observed that SerpinB2-expressing tumor cells showed reduced migration and length of invadopodia-like structures (Fig. 4), an activity likely mediated by inhibition of uPA 13–15,27. These data are consistent with the classical view that tumor-derived SerpinB2 inhibits uPA-mediated migration and invasion, thereby inhibiting metastasis 14,32.

The controversy surrounding the physiological function of SerpinB2 is in part due to the inefficient secretion of SerpinB2, making it hard to understand how efficient inhibition of extracellular uPA could occur 1. A key observation made herein is that tumor-expressed SerpinB2 is present on the surface of tumor-derived MPs (Fig. 5). Tumor cells, including B16 36, are known to generate considerable number of MPs 34, with such MPs known to play key roles in tumor invasion and metastasis 36,46. Specifically, MPs have been shown to contain uPA, with MP-associated uPA promoting cancer cell invasion 47,48. MP-associated uPA is also believed to be important for metastasis 35. Herein we observed uPA on tumor-derived MPs (Fig. 5B) as well as reduced migration and invasion (Fig. 4) and metastasis (Fig 3) for SerpinB2-expressing tumor cells. The clear suggestion is that SerpinB2 associated with tumor-derived MPs inhibits MP-associated uPA, thereby inhibiting tumor cell migration, invasion, and metastasis.

The finding that SerpinB2 is expressed on MPs (herein and 33) begs the question of how SerpinB2 might be retained on the surface of MPs? The evidence presented herein suggests annexins mediate binding of SerpinB2 to PS (Fig. 6). PS is present on the surface of MPs 37,39 and annexins are found on MPs 42,43. Thus, although SerpinB2 has been shown to bind annexins 40 and annexins are known to bind PS 49, we show herein for the first time that annexin mediates the association of SerpinB2 with PS. This provides a simple model for understanding how SerpinB2 might be retained on the surface of MPs.

Given the association of SerpinB2 with annexins and PS, it is tempting to speculate that SerpinB2 is somehow externalized with annexin/PS during MP formation. Both annexins and SerpinB2 have been reported to be secreted via (poorly understood) nonclassical or unconventional pathways 4,50. Increased cytoplasmic calcium concentrations trigger MP formation and binding of annexins to PS 49. PS is then believed to be translocated to the outer membrane with the help of transporters and translocators (e.g., floppase and scramblase) 37. The rapid externalization of SerpinB2 during MP formation (Fig. 5D) would provide a novel mechanism for SerpinB2 secretion, and may also provide important insights into how and when SerpinB2 accesses the extracellular milieu.

The presence of SerpinB2 on MPs may also be a key to unraveling the disparate activities reported for SerpinB2 1. MPs control a large spectrum of activities in inter alia cancer, inflammation and apoptosis, with the cellular source of the MPs often determining their activities 34,39,46. Herein, we provide evidence that at least one of the activities of SerpinB2 on cancer-derived MPs is to suppress uPA-mediated tumor cell migration, invasion, and metastasis.

## Supporting Information

**Figure S1.** Anti-cancer CD8+ T-cell responses in SerpinB2^−/−^ and SerpinB2^+/+^ mice. ELISPOT analysis of splenocytes obtained from SerpinB2^−/−^ and SerpinB2^+/+^ mice 18 days after i.v. injection of B16 cells. The assay used the peptides KVPRNQDWL from gp100 and SVYDFFVWL from Trp2 17. The ex vivo and cultured ELISPOT assays are a measure of effector and memory CD8^+^ T-cell responses, respectively (Rattanasena et al., Immunol. Cell Biol. 2011;89:426–436) (*n* = 8 mice per group). **Figure S2.** mRNA expression levels of selected genes in B16 tumors grown in SerpinB2^−/−^ or SerpinB2^+/+^ mice. Tumors were surgically dissected when they reached ≈100 mm^2^ and nontumor tissue was removed as much as possible. mRNA levels were determined by quantitative real time RT-PCR normalized against RPL13A mRNA as described [12, 23] (*n* = 8–10 per group, except IL-6 where *n* = 2, analyzed in duplicate). Primers for TGF-*β* were F 5′-TGACGTCACTGGAGTTGTACGG-3′, R: 5′-GGTTCATGTCATGGATGGTGC-3′ and for mouse uPA were F 5′-TGCCCAAGGAAATTCCAGGG-3′, R: 5′-GCCAATCTGCACATAGCACC-3′. **Figure S3.** qRT-PCR of SerpinB2 expression. SerpinB2 mRNA expression levels were determined in the indicated cells grown in vitro by quantitative real time RT-PCR normalized against RPL13A mRNA levels as described 12. (Two cultures analyzed in duplicate.) RPM, resident peritoneal macrophages cultured for 2 days. B16- and Tubo-SerpinB2 cells express ≈30% of the levels of SerpinB2 mRNA that are constitutively expressed in RPM from SerpinB2^+/+^ mice. **Figure S4.** Effect of SerpinB2 expression on cell proliferation as determined by anti-Ki67 staining. Tubo tumors (100 mm^2^) were fixed in paraformaldehyde and paraffin sections stained with anti-Ki67 (as described Cozzi et al. J. Invest. Dermatol. 2012;132:1263–1271). The percentage of Ki67+ nuclei was determined using Aperio IHC nuclear image analysis algorithm. *N* = 3 tumors per group, two sections per tumor were analyzed. **Figure S5.** qRT-PCR of SerpinB2 expression in B16-SerpinB2 and B16-Control tumors grown in SerpinB2^+/+^ or SerpinB2^−/−^ mice. B16-SerpinB2 and B16-Control tumors grown in SerpinB2^+/+^ or SerpinB2^−/−^ mice were surgically removed when they reached ≈100 mm^2^ and nontumor tissue was removed as much as possible. SerpinB2 mRNA expression levels were determined by quantitative real time RT-PCR normalized against RPL13A mRNA as described 12. The expression of SerpinB2 mRNA was retained in B16-SerpinB2-derived tumors; see mRNA levels in B16-SerpinB2 tumors (grown in either SerpinB2^+/+^ or SerpinB2^−/−^ mice). **Figure S6.** In vitro growth of B16 and Tubo lines described in Figure 2. Cell protein levels were determined using crystal violet staining at the indicated times as described (Antalis et al. J. Exp. Med. 1998;187;1799–1811). Briefly, parallel cultures in triplicate (in 96 well plate format) were fixed and stained with crystal violet at the indicated times, washed and OD measured after dissolving the retained dye in methanol. **Figure S7.** Effect of SerpinB2 expression on in vitro growth of human tumor cell lines. Three tumor cell lines were transduced with lentiviral vectors encoding EGFP (EGFP), SerpinB2 (SerpinB2) or the CD interhelical mutant of SerpinB2 (ΔCD) 18. (A) The transduced lines and the parental lines were then tested for expression of SerpinB2 or CD interhelical mutant of SerpinB2 by immunoblotting (B) The same lines were then assayed for growth using the crystal violet assay (as described above). (C) The lentivirus transduced A549 lines described above were stained with PI and cell cycle profile determined using FACSCalibur flow cytometer (Becton Dickinson) using CellQuest Pro (Becton Dickinson) and analyzed using Modfit (Verity Software House Inc.) software. **Figure S8.** Cell cycle profiles in two cancer cell lines following SerpinB2 expression by transient transfection. (A) A549 and SAOS-2 cells were cotransfected (GeneJammer) with empty plasmid or the same plasmids expressing human SerpinB2, SerpinB2-ΔCD (Dickinson et al. J. Biol. Chem. 1995;270:27894–27904) in combination with a plasmid encoding EGFP (Clontech). After 48 h cells were stained with propidium iodide and cell cycle profiles of EGFPhi cells determined using FACSCalibur flow cytometer (Becton Dickinson) using CellQuest Pro (Becton Dickinson) and analyzed using Modfit (Verity Software House Inc) software (*n* = 2) (∼40–50% of cells were EGFP positive). (B) Transient transections results in SerpinB2 expression as shown by Western analysis (see also 11). **Figure S9.** In vitro growth of MEFs from SerpinB2^+/+^ and SerpinB2^−/−^mice. (A) Growth of spontaneously immortalized murine embryonic fibroblasts (MEFs) (with and without 100 ng/mL LPS) as determined by crystal violet staining (Antalis et al. J. Exp. Med. 1998;187:1799–1811) at the indicated times. (B) Western analysis of MEFs from SerpinB2^+/+^ and SerpinB2^−/−^mice. (C) LPS-induced SerpinB2 mRNA expression (normalized against RPL13A mRNA expression) in MEFs from SerpinB2^+/+^ mice. **Figure S10.** In vitro growth of thioglycollate-elicited peritoneal macrophages (TEPMs) from SerpinB2^+/+^ and SerpinB2^−/−^mice. (A) TEPMs were cultured for 9 days in the absence or presence of LPS (100 ng/mL). At each time point parallel cell cultures were trypsinized and counted using trypan blue. (B) Western analysis of SerpinB2 expression in TEPMs using a goat polyclonal anti-human rPAI-2 antibody (kind gift from Drs. M. Wilczynska and T. Ny (Umeå University, Umeå, Sweden). **Figure S11.** uPA protein expression in B16 lines. Western blot analysis of murine uPA in lysates of B16-SerpinB2 and B16-Control cell lines. **Figure S12.** Actin staining showing invadopodia-like structures in B16-SerpinB2 and B16-Control cells. Repeat of experiment shown in Figure 4C, except cells were plated into Matrigel on glass coverslips and were cultured for 24 h, followed by fixation in 2% paraformaldehyde 0.1% Triton X-100 in PBS, washing, staining with phalloidin-rhodamine (a stain for actin present in invadopodia; Ridley Cell 2011;145:1012–1022) and viewing by confocal microscopy. Mean length of invadopodia-like structures per cell for B16-SerpinB2 cells was 6.2 + 0.6 *μ*m SE, for B16-Control it was 1.9 + 0.31 *μ*m SE (*P* < 0.001, Kolmogorov–Smirnov test). **Figure S13.** Densitometry of SerpinB2 lipid-binding experiments. Scanning densitometry of three independent repeat experiments of the experiment described in Figure 6. Densitometry was performed using ImageQuant TL Array v8.1. Background was subtracted using spot edge average.
