# Supplementary material for: Tumor cell-expressed SerpinB2 is present on microparticles and inhibits metastasis
Source: Cancer Med. 2014 Mar 19;3(3):500–13. doi: 10.1002/cam4.229 (PMC4101741; doi:10.1002/cam4.229)
Supplement: Supplementary file 1 — Figure S1. Anti-cancer CD8+ T-cell responses in SerpinB2−/− and SerpinB2+/+ mice. ELISPOT analysis of splenocytes obtained from SerpinB2−/− and SerpinB2+/+ mice 18 days after i.v. injection of B16 cells. The assay used the peptides KVPRNQDWL from gp100 and SVYDFFVWL from Trp2 17. The ex vivo and cultured ELISPOT assays are a measure of effector and memory CD8+ T-cell responses, respectively (Rattanasena et al., Immunol. Cell Biol. 2011;89:426–436) (n = 8 mice per group). Figure S2. mRNA expression levels of selected genes in B16 tumors grown in SerpinB2−/− or SerpinB2+/+ mice. Tumors were surgically dissected when they reached ≈100 mm2 and nontumor tissue was removed as much as possible. mRNA levels were determined by quantitative real time RT-PCR normalized against RPL13A mRNA as described [12, 23] (n = 8–10 per group, except IL-6 where n = 2, analyzed in duplicate). Primers for TGF-β were F 5′-TGACGTCACTGGAGTTGTACGG-3′, R: 5′-GGTTCATGTCATGGATGGTGC-3′ and for mouse uPA were F 5′-TGCCCAAGGAAATTCCAGGG-3′, R: 5′-GCCAATCTGCACATAGCACC-3′. Figure S3. qRT-PCR of SerpinB2 expression. SerpinB2 mRNA expression levels were determined in the indicated cells grown in vitro by quantitative real time RT-PCR normalized against RPL13A mRNA levels as described 12. (Two cultures analyzed in duplicate.) RPM, resident peritoneal macrophages cultured for 2 days. B16- and Tubo-SerpinB2 cells express ≈30% of the levels of SerpinB2 mRNA that are constitutively expressed in RPM from SerpinB2+/+ mice. Figure S4. Effect of SerpinB2 expression on cell proliferation as determined by anti-Ki67 staining. Tubo tumors (100 mm2) were fixed in paraformaldehyde and paraffin sections stained with anti-Ki67 (as described Cozzi et al. J. Invest. Dermatol. 2012;132:1263–1271). The percentage of Ki67+ nuclei was determined using Aperio IHC nuclear image analysis algorithm. N = 3 tumors per group, two sections per tumor were analyzed. Figure S5. qRT-PCR of SerpinB2 expression in B16-SerpinB2 and B16-Control [file cam40003-0500-SD1.pdf]

Supporting Information

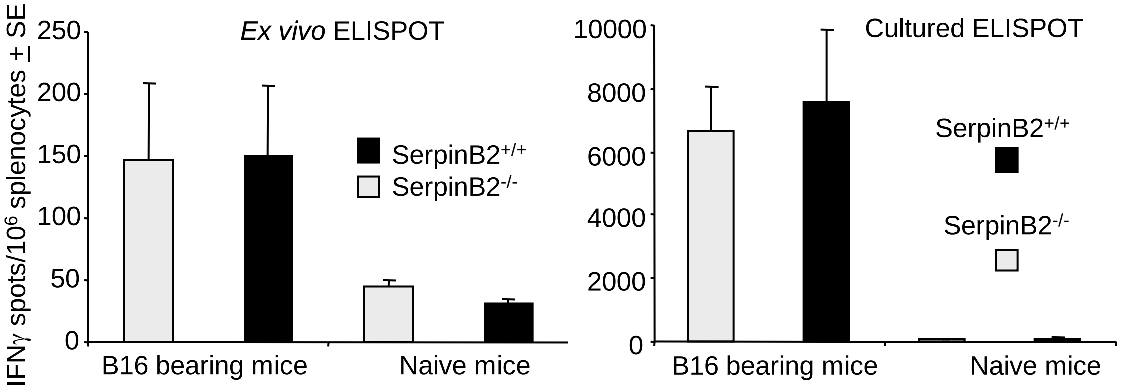

Figure 1. **Anti-cancer CD8+ T cell responses in SerpinB2$^{-/-}$ and SerpinB2$^{+/+}$ mice**. ELISPOT analysis of splenocytes obtained from SerpinB2$^{-/-}$ and SerpinB2$^{+/+}$ mice 18 days after i.v. injection of B16 cells. The assay used the peptides KVPRNQDWL from gp100 and SVYDFFVWL from Trp2 (Le et al. 2009. Vaccine 27; 3053-62). The *ex vivo* and cultured ELISPOT assays are a measure of effector and memory CD8^{+} T cell responses, respectively (Rattanasena et al., 2011. Immunol Cell Biol 89;426-436). (n=8 mice per group).

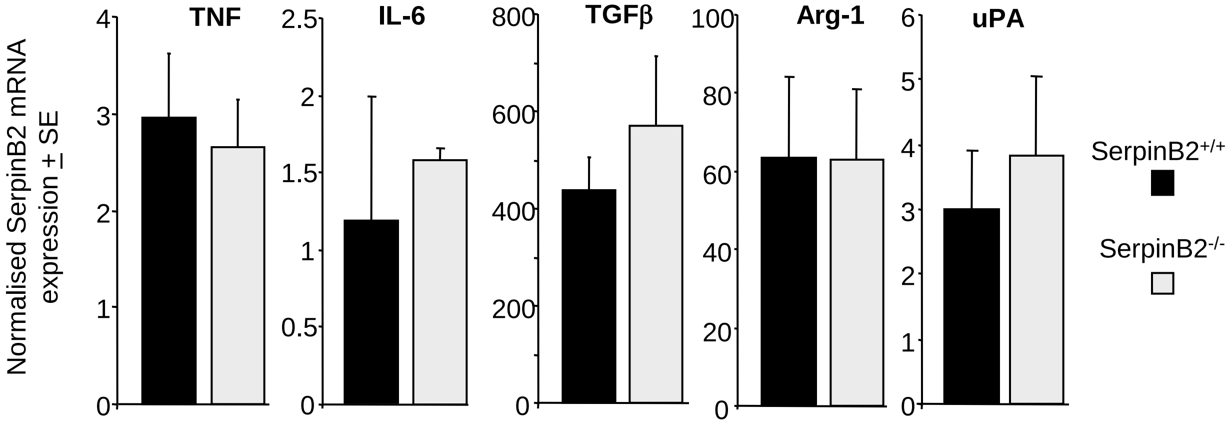

Figure 2. mRNA expression levels of selected genes in B16 tumors grown in SerpinB2$^{-/-}$ or SerpinB2$^{+/+}$ mice. Tumors were surgically dissected when they reached ≈100 mm^2 and non-tumor tissue was removed as much as possible. mRNA levels were determined by quantitative real time RT-PCR normalised against RPL13A mRNA as described (Schroder et al., 2010. J Immunol. 184; 2663-2670; Schroder et al., 2012. Parasite Immunol 32; 764-8). (n=8-10 per group, except IL-6 where n=2, analyzed in duplicate). Primers for TGFβ were F 5'- TGACGTCACTGGAGTTGTACGG -3‘, R: 5'- GGTTCATGTCATGGATGGTGC -3' and for mouse uPA were F 5'- TGCCCAAGGAAATTCCAGGG -3‘, R: 5'- GCCAATCTGCACATAGCACC -3'

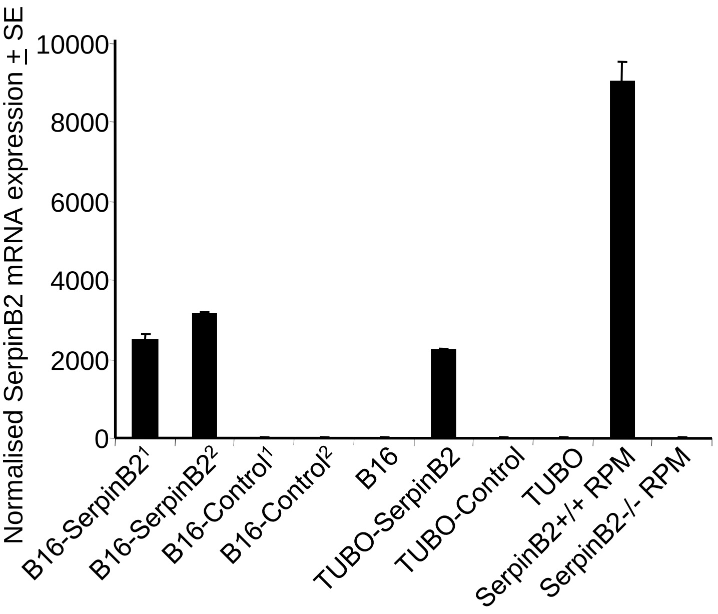

Figure 3. **qRT-PCR of SerpinB2 expression.** SerpinB2 mRNA expression levels were determined in the indicated cells grown *in vitro* by quantitative real time RT-PCR normalised against RPL13A mRNA levels as described (Schroder et al., 2010. J Immunol. 184; 2663-2670). (Two cultures analyzed in duplicate). RPM –resident peritoneal macrophages cultured for 2 days. B16- and TUBO-SerpinB2 cells express ≈30% of the levels of SerpinB2 mRNA that are constitutively expressed in RPM from SerpinB2$^{+/+}$ mice.

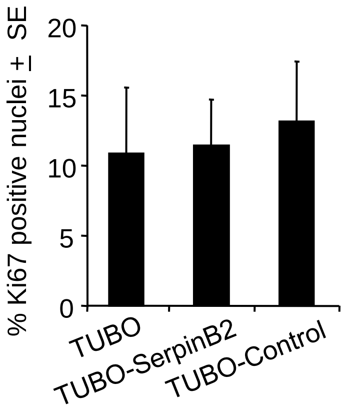

Figure 4. **Effect of SerpinB2 expression on cell proliferation as determined by anti-Ki67 staining.** TUBO tumors (100mm^2) were fixed in paraformaldehyde and paraffin sections stained with anti-Ki67 (as described Cozzi et al., J Invest Dermatol. 2012. 132(4):1263-71). The percentage of Ki67$^+$ nuclei was determined using Aperio IHC nuclear image analysis algorithm. N= 3 tumors per group, 2 sections per tumor were analyzed.

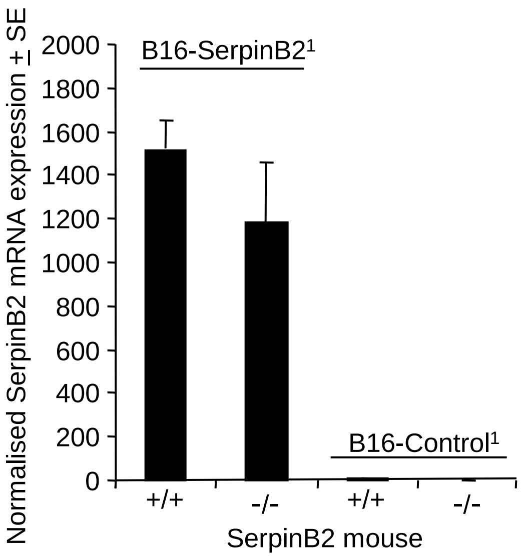

Figure 5. **qRT PCR of SerpinB2 expression in B16-SerpinB2 and B16-Control tumors grown in SerpinB2$^{+/+}$ or SerpinB2$^{-/-}$ mice.** B16-SerpinB2 and B16-Control tumors grown in SerpinB2$^{+/+}$ or SerpinB2$^{-/-}$ mice were surgically removed when they reached ≈100 mm^2 and non-tumor tissue was removed as much as possible. SerpinB2 mRNA expression levels were determined by quantitative real time RT-PCR normalised against RPL13A mRNA as described (Schroder et al., 2010. J Immunol. 184; 2663-2670).

The expression of SerpinB2 mRNA was retained in B16-SerpinB2 derived tumors; see mRNA levels in B16-SerpinB2 tumors (grown in either SerpinB2$^{+/+}$ or SerpinB2$^{-/-}$ mice).

Figure 6. *In vitro* growth of B16 and TUBO lines described in Fig. 2. Cell protein levels were determined using crystal violet staining at the indicated times as described (Antalis et al., 1998. 187;1799-1811). Briefly, parallel cultures in triplicate (in 96 well plate format) were fixed and stained with crystal violet at the indicated times, washed and OD measured after dissolving the retained dye in methanol.

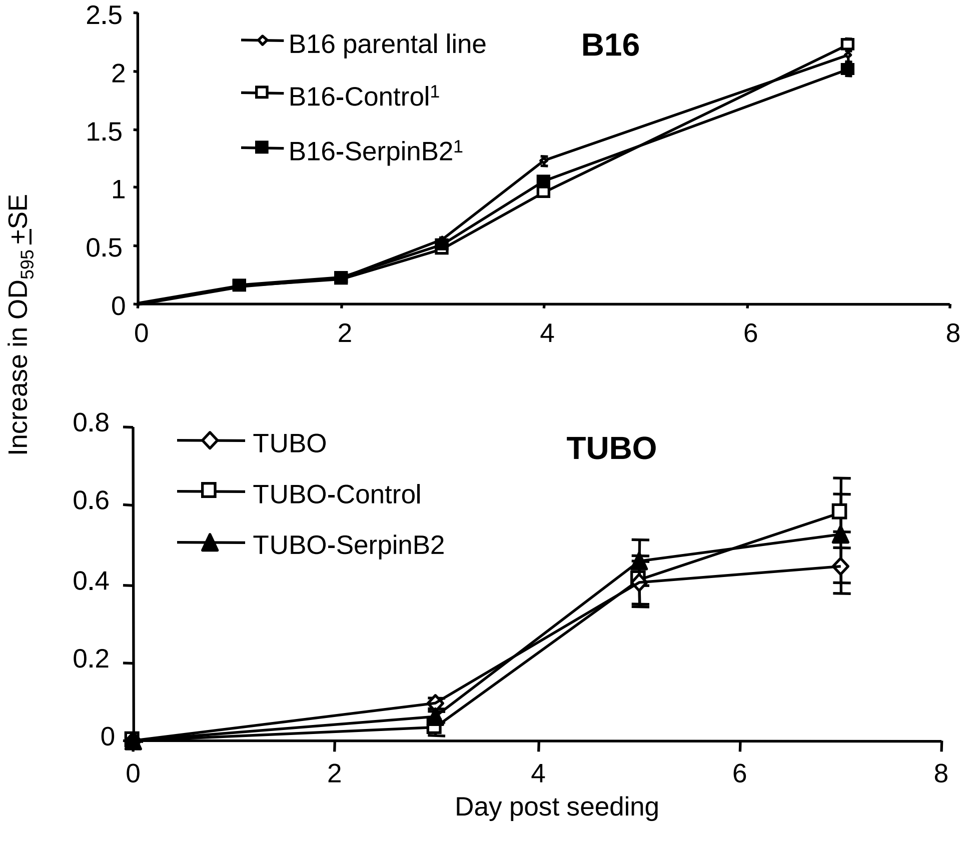

Figure 7. **Effect of SerpinB2 expression on *in vitro* growth of human tumor cell lines.** Three tumor cell lines were transduced with lentiviral vectors encoding EGFP (EGFP), SerpinB2 (SerpinB2) or the CD interhelical mutant of SerpinB2 (ΔCD) (Fish & Kruithof, 2006 Exp Cell Res 312; 350-61). (A) The transduced lines and the parental lines were then tested for expression of SerpinB2 or CD interhelical mutant of SerpinB2 by immunoblotting (B) The same lines were then assayed for growth using the crystal violet assay (as described above).

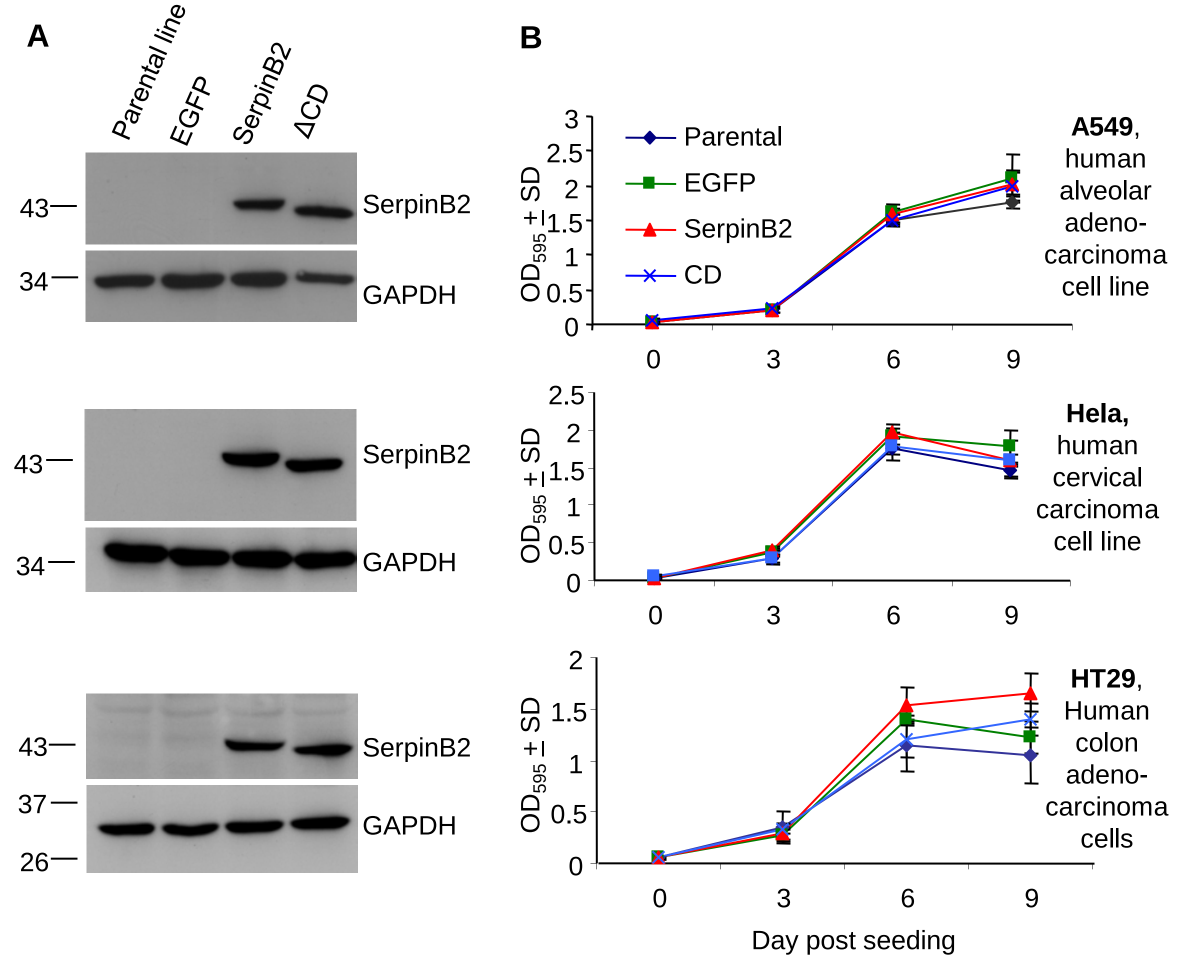

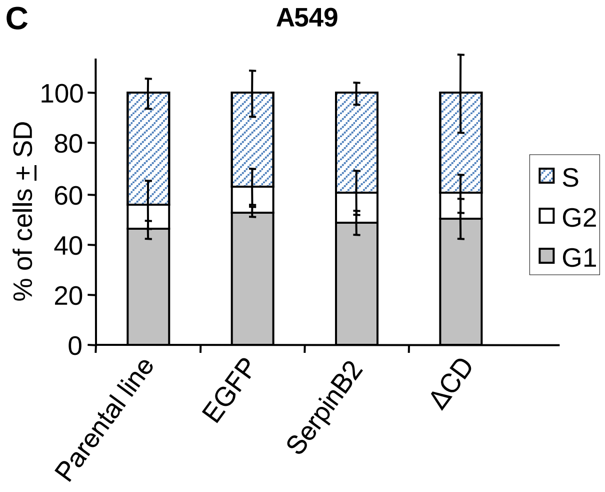

(C) The lentivirus transduced A549 lines described above were stained with PI and cell cycle profile determined using FACSCalibur flow cytometer (Becton Dickinson) using CellQuest Pro (Becton Dickinson) and analysed using Modfit (Verity Software House Inc) software.

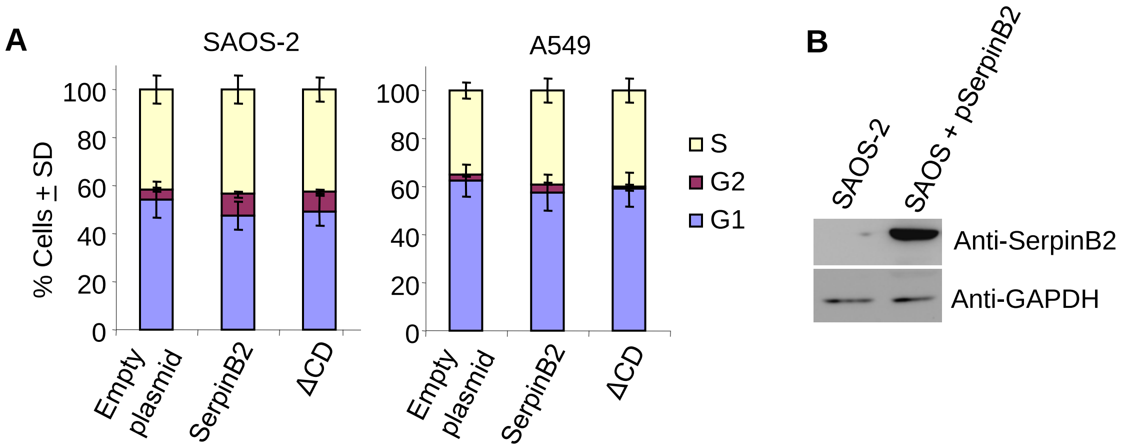

Figure 8. Cell cycle profiles in two cancer cell lines following SerpinB2 expression by transient transfection. (A) A549 and SAOS-2 cells were co-transfected (GeneJammer) with empty plasmid or the same plasmids expressing human SerpinB2, SerpinB2-ΔCD (Dickinson et al. 1995. J Biol Chem. 270:27894-904) in combination with a plasmid encoding EGFP (Clontech). After 48 h cells were stained with propidium iodide and cell cycle profiles of $EGFP^{hi}$ cells determined using FACSCalibur flow cytometer (Becton Dickinson) using CellQuest Pro (Becton Dickinson) and analysed using Modfit (Verity Software House Inc) software (n=2). (Approximately 40-50% of cells were EGFP positive). (B) Transient transections results in SerpinB2 expression as shown by Western analysis (see also Yu et al. 2002. Blood 99; 2810-2818).

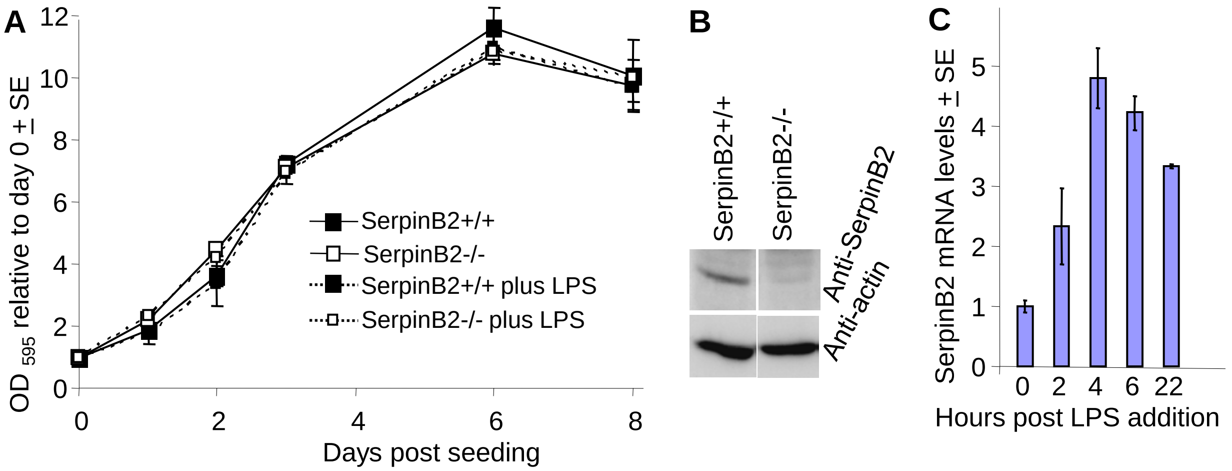

Figure 9. ***In vitro* growth of MEFs from SerpinB2$^{+/+}$ and SerpinB2$^{-/-}$ mice.** (A) Growth of spontaneously immortalized murine embryonic fibroblasts (MEFs) (with and without 100 ng/ml LPS) as determined by crystal violet staining (Antalis et al., 1998. 187;1799-1811) at the indicated times. (B) Western analysis of MEFs from SerpinB2$^{+/+}$ and SerpinB2$^{-/-}$ mice. (C) LPS-induced SerpinB2 mRNA expression (normalised against RPL13A mRNA expression) in MEFs from SerpinB2$^{+/+}$ mice.

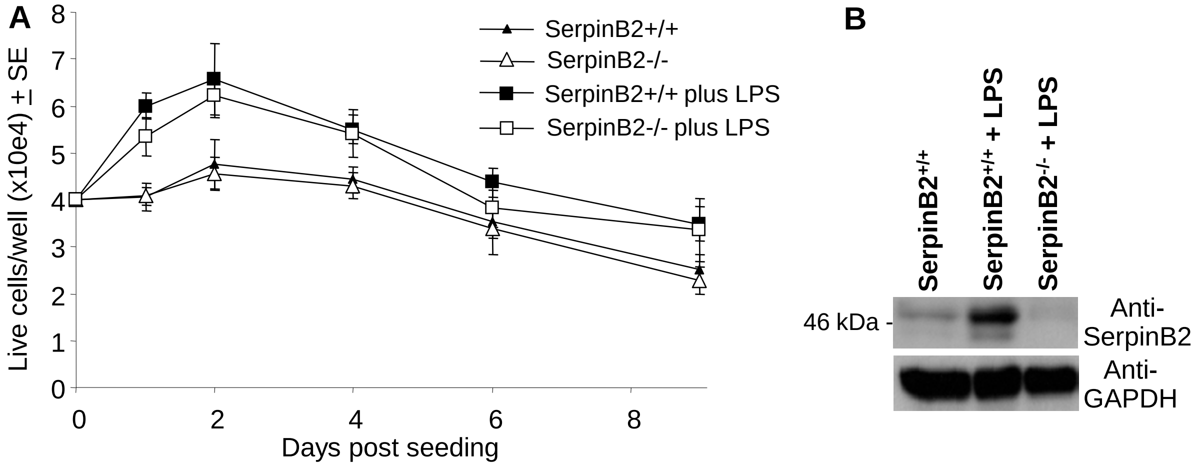

Figure 10. *In vitro* growth of thioglycollate-elicited peritoneal macrophages (TEPMs) from SerpinB2$^{+/+}$ and SerpinB2$^{-/-}$ mice. (A) TEPMs were cultured for 9 days in the absence or presence of LPS (100 ng/ml). At each time point parallel cell cultures were trypsinized and counted using trypan blue. (B) Western analysis of SerpinB2 expression in TEPMs using a goat polyclonal anti-human rPAI-2 antibody (kind gift from Drs M Wilczynska and T Ny (Umeå University, Umeå, Sweden).

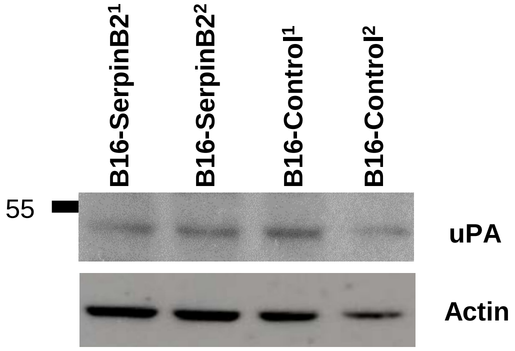

Figure 11. **uPA protein expression in B16 lines**. Western blot analysis of murine uPA in lysates of B16-SerpinB2 and B16-Control cell lines.

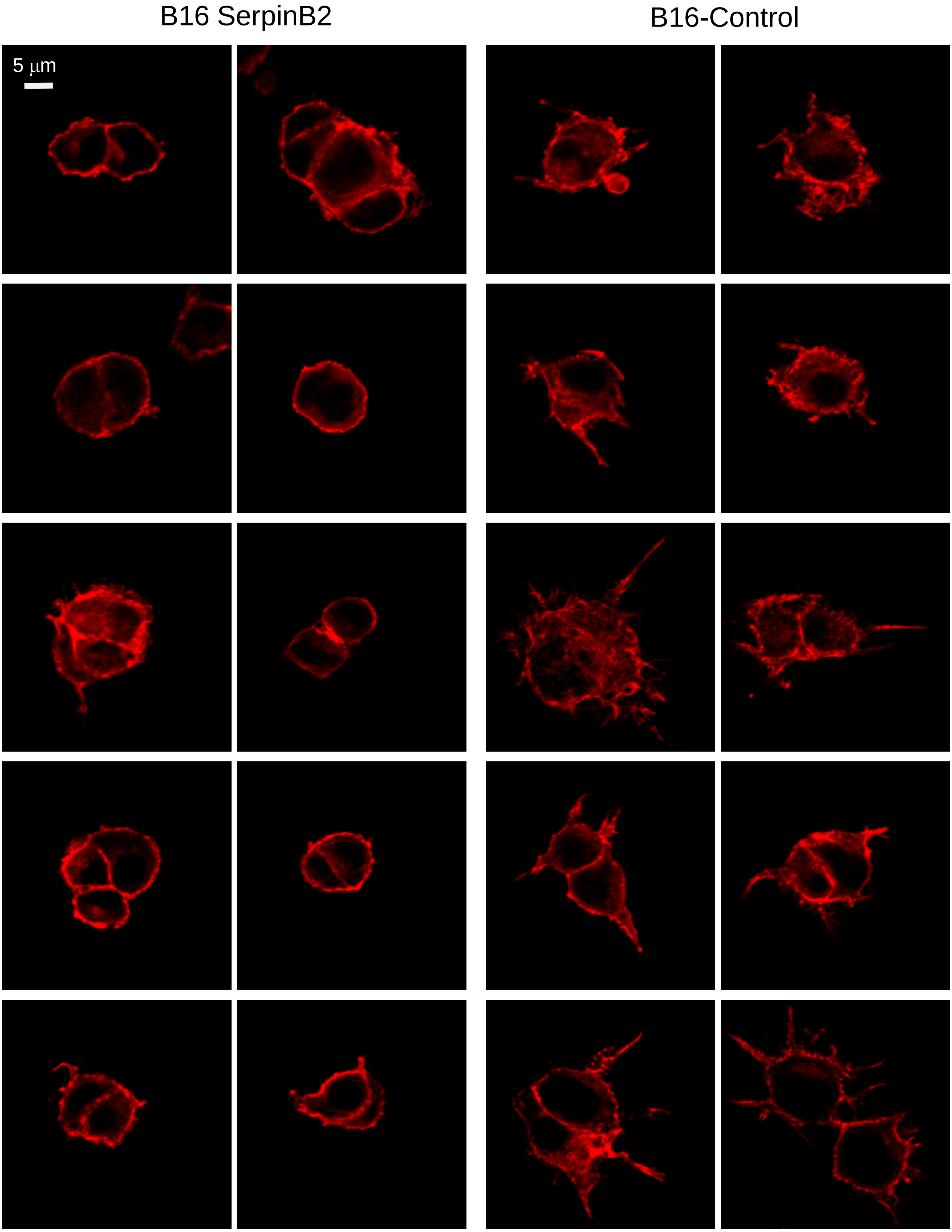

Figure 12. Actin staining showing invadopodia-like structures in B16-SerpinB2 and B16-Control cells. Repeat of experiment shown in Fig. 4C, except cells were plated into Matrigel on glass coverslips and were cultured for 24 h, followed by fixation in 2% paraformaldehyde 0.1% Triton X100 in PBS, washing, staining with phalloidin-rhodamine (a stain for actin present in invadopodia; Ridley 2011 Cell 145;1012-22.) and viewing by confocal microscopy. Mean length of invadopodia-like structures per cell for B16-SerpinB2 cells was 6.2 µm ± 0.6 SE, for B16-Control it was 1.9 µm ± 0.31 SE ($p<0.001$, Kolmogorov-Smirnov test).

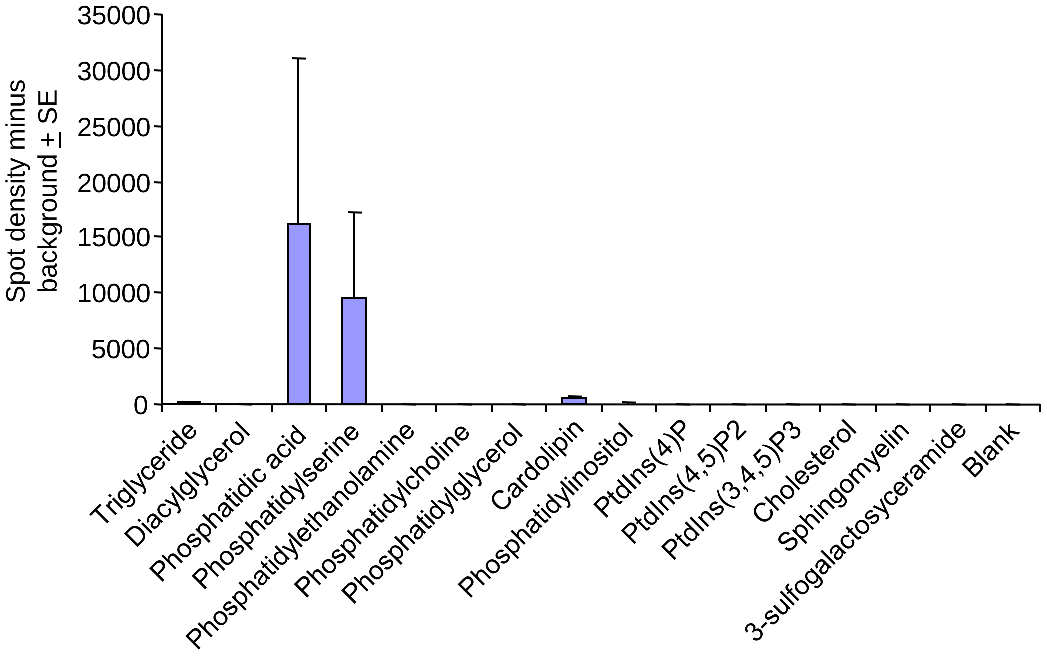

Figure 13. Densitometry of SerpinB2 lipid binding experiments. Scanning densitometry of three independent repeat experiments of the experiment described in Fig. 7. Densitometry was performed using ImageQuant TL Array v8.1. Background was subtracted using spot edge average.
